# Roles of PknB and CslA in Cell Wall Morphogenesis of *Streptomyces*

**DOI:** 10.1111/mmi.70086

**Published:** 2026-06-09

**Authors:** Marta Derkacz, Andrew Watson, Akshada Gajbhiye, Michał Tracz, Dagmara Jakimowicz, Matthias Trost, Jeff Errington, Bernhard Kepplinger

**Affiliations:** ^1^ Department of Molecular Microbiology University of Wroclaw Wrocław Poland; ^2^ Centre for Bacterial Cell Biology Newcastle University Newcastle upon Tyne UK; ^3^ Faculty of Medical Sciences Newcastle University Biosciences Institute Newcastle upon Tyne UK; ^4^ Mass Spectrometry Laboratory University of Wroclaw Wrocław Poland; ^5^ Faculty of Medicine and Health The University of Sydney Sydney New South Wales Australia

**Keywords:** cell morphogenesis, cell wall, CslA, DivIVA, phosphorylation, PknB, polarisome, *Streptomyces*, Tip Organising Centre (TIPOC)

## Abstract

The bacterial cell wall is essential for maintaining cellular integrity and defining the mode of growth, with different species adopting distinct strategies for cell wall synthesis and remodelling. *Streptomyces* are filamentous bacteria predominantly found in soil and renowned for producing specialised metabolites, including antibiotics. They grow through tip extension and branching hyphal filaments, forming a multicellular mycelium. New branches are established by forming a new growth zone on the lateral cell wall. Proteins involved in this process are organised into complexes called polarisomes, with DivIVA being the most well‐characterised component. To investigate the tip growth requirements in *Streptomyces albus* we developed a genetic screen utilising toxic DivIVA overproduction and searched for suppressors of its lethality, reasoning that such suppressors would likely encode components functionally linked to DivIVA or the tip growth machinery. Among the identified genes was *pknB*, encoding a serine/threonine protein kinase implicated in the regulation of cell growth and morphogenesis. We confirmed that deletion of *pknB* restored the growth phenotype of *S. albus* following DivIVA overproduction. The phosphoproteome analysis revealed that the absence of PknB alters the phosphorylation state of CslA, a cellulose synthase that synthesizes a beta‐glucan (cellulose) polymer. We demonstrate that a phosphoablative mutant of CslA impairs beta‐glucan synthesis and causes hypersensitivity to lysozyme. Overproduction of CslA restored colony growth defects arising from DivIVA‐induced hyperbranching, without however suppressing the hyperbranching phenotype. These findings collectively identify PknB‐dependent phosphorylation of CslA as a central regulatory point in *Streptomyces* cell envelope construction, revealing how modulation of beta‐glucan synthesis can mitigate the cellular consequences of DivIVA dysregulation.

## Graphical abstract

*Streptomyces* grow by extending hyphal tips, a process regulated by polarisome complexes anchored by DivIVA. Using a genetic screen based on toxic DivIVA overproduction, we identified the kinase PknB as a key regulator of cell envelope construction. PknB‐dependent signalling controls the phosphorylation state of CslA, a β‐glucan synthase, and this modification buffers cells against the harmful effects of DivIVA dysregulation.

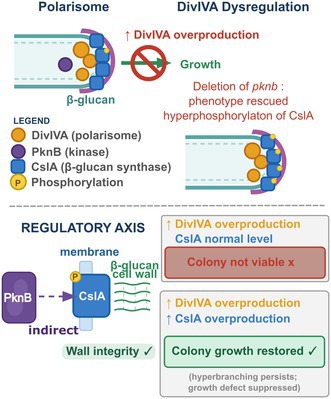

## 1 Introduction

Peptidoglycan, a major target for antibiotics, is an essential component of bacterial cell walls, providing structural integrity and rigidity and enabling bacteria to maintain their shape and withstand osmotic pressure challenges (Liu and Breukink 2016; Garde et al. 2021). Proteobacteria (e.g., *Escherichia coli* ) and Firmicutes (e.g., *Bacillus subtilis* ) are among the best‐studied models for cell morphogenesis. These rod‐shaped bacteria typically exhibit lateral insertion of new cell wall material during elongation (Egan et al. 2020). In contrast, Actinobacteria (e.g., *Streptomyces* and *Mycobacteria*) mainly insert new cell wall material at the tips during exponential growth (Flärdh 2003a; Kuru et al. 2014; Zambri et al. 2025). Actinobacteria can be unicellular, such as Mycobacteria or Corynebacteria, or multicellular (e.g., *Streptomyces*), the latter forming a complex mycelial network that supports substrate colonisation (Chater 2006). This polar growth mode is hypothesised to confer ecological advantages, such as efficient nutrient foraging and competitive dominance in soil environments.

Cell wall biosynthetic proteins driving tip growth are organised locally into polarisome complexes (Hempel et al. 2008). Within the polarisome, DivIVA—a coiled‐coil protein—plays a pivotal role and is essential for *Streptomyces* growth (Flärdh 2003b). Recent studies have further refined our understanding of DivIVA domain function, specifically how its N‐terminal membrane‐binding and C‐terminal oligomerization domains coordinate to maintain a curvature‐sensitive scaffold (Lubbers et al. 2025). Overproduction of DivIVA results in swollen tips and hyperbranching, likely due to the misregulation of the cell wall biosynthetic machinery (Flärdh 2003b). New DivIVA complexes appear on the lateral wall preceding the formation of the new branches (Flärdh 2003b; Hempel et al. 2008; Richards et al. 2012). Two additional scaffolding proteins, Scy and FilP, are believed to sequester DivIVA, thereby facilitating the establishment of new growth zones and influencing the size and position of DivIVA complexes, respectively (Holmes et al. 2013; Fröjd and Flärdh 2019). The precise mechanism for branch initiation remains, however, elusive (Hempel et al. 2008).

DivIVA phosphorylation has been reported in a wide range of species (Kang et al. 2005; Beilharz et al. 2012; Elsholz et al. 2012). *Streptomyces coelicolor* encodes at least 34 Ser/Thr kinases, and DivIVA is modified by the non‐essential Ser/Thr kinase AfsK (Hempel et al. 2012). Mass spectrometry has identified AfsK phosphorylation sites on DivIVA as occurring on the non‐essential C‐terminal protein at residues T304, S309, S338, S344, and S355 in *S. coelicolor* (Saalbach et al. 2013). The phosphatase SppA appears to function as a counterpart in this process (Passot et al. 2022). Notably, AfsK overexpression induces disassembly of the apical (mother) polarisome, leading to the formation of multiple daughter polarisomes, followed by the establishment of multiple hyphal branches (Hempel et al. 2008).

In contrast, in Mycobacteria, the DivIVA homologue Wag31 is phosphorylated by the essential kinases PknA and PknB (Jani et al. 2010), which are themselves encoded alongside a rodA‐pbp pair within a conserved gene cluster (Molle and Kremer 2010; Iswahyudi et al. 2019). Interestingly, the elimination of the homologous kinase PknB in *Streptomyces* has no detrimental effect, whereas the kinase PknA is missing in most *Streptomyces* spp. (Jones et al. 2011).

In this study, we investigated the consequences of DivIVA overproduction in *Streptomyces albus* to uncover the factors that are involved in tip growth. This study revealed that inactivation of the kinase PknB unexpectedly alleviated defects resulting from DivIVA imbalance. Subsequent analysis identified CslA as a key downstream target of PknB, a cellulose synthase protein that synthesises β‐glucan and contributes to cell wall biogenesis (Xu et al. 2008). We further demonstrate that CslA phosphorylation state modulates β‐glucan synthesis and, consequently, the cell's ability to stabilise growth zones. These findings suggest a phosphorylation‐dependent regulatory mechanism linking PknB activity to cell wall assembly at hyphal tips.

## 2 Materials and Methods

### 2.1 Bacterial Strains and Culture Conditions

All *S. albus* strains used in this study are listed in Table S1. *Streptomyces albus* spores were collected using standard protocols (Kieser et al. 2000) from cultures grown on Soya Flour Mannitol (SFM) agar plates supplemented with antibiotics (if required) to a final concentration of apramycin (50 μg/mL) and hygromycin (50 μg/mL). Spore concentration was determined by serial dilution on SFM agar plates. Spores were maintained at –80°C in 25% glycerol aliquots for single‐use. Tryptic Soy Broth (TSB) and 5% TSB were used for morphology experiments. Liquid cultures were performed in 250 mL Erlenmeyer flasks containing metal spirals at 30°C and 220 rpm for agitation. Solid cultures were grown on 20 mL of 2% or 5% TSA agar plates at 30°C. *Escherichia coli* strains (listed in Table S2) were grown at 37°C on LB supplemented with antibiotics (if required) to a final concentration of apramycin (50 μg/mL), kanamycin (50 μg/mL), chloramphenicol (34 μg/mL), or on 2YT supplemented with hygromycin (50 μg/mL).

### 2.2 Construction of Plasmids and Strains

All plasmids and oligonucleotides used in this study are listed in Tables S3 and S4, respectively. Details of the plasmid construction are provided in Methods S1, specifically in the section on comprehensive plasmid design and assembly. Plasmids were transformed into *E. coli* DH5α and subsequently transferred to *Streptomyces* by conjugation with *E. coli* ET12567/pUZ8002 strain or protoplast transformation using standard protocols (Kieser et al. 2000). For integrative plasmids, *Streptomyces* strains were grown in the presence of the appropriate antibiotics. CRISPR/Cas9 plasmids were cured by incubation at 42°C without antibiotics. Mutants lacking apramycin resistance were sequenced by Microsynth AG using PCR‐amplified fragments of modified genomic DNA (with the exception of the 65α, 65α attBϕC31:: pIJ6902 (empty plasmid), and 65α attBϕC31::pBK288 (CslA under *tipA* inducible promoter) strains, which were verified by Illumina sequencing).

### 2.3 Selection of Spontaneous Suppressor Mutants and Bioinformatics Analysis

Spores of the DivIVA overproduction strain (approximately 10^6^ spores per plate) were spread onto 5% TSA plates supplemented with 500 ng/mL of inducer (anhydrotetracycline). Following 5 days of incubation, several colonies were observed and subsequently restreaked onto fresh 5% TSA containing 500 ng/mL of the inducer (anhydrotetracycline). The parent DivIVA‐overproducing and wild‐type strains served as controls.

DNA was isolated from the strains using a modified version of the “salting‐out” method (Pospiech and Neumann 1995; Watson et al. 2022). Libraries for Illumina sequencing were prepared using the Illumina DNA prep kit, Nextera XT kit, and Illumina Nextera XT primer Index Kit. Sequencing was performed by Edinburgh Genomics using MiSeq v2 (250 PE). Raw sequences are available in the NCBI Sequence Read Archive (accession number: PRJNA1099611).

The quality of all Illumina reads was assessed using fastQC (version 0.11.9), and the reads were trimmed using trimmomatic (version 0.39). Bowtie2 (version 2.3.5.1) was used to align the trimmed Illumina reads to the *Streptomyces albus* J1074 genome (NC_020990.1) using the very sensitive preset mode for mapping paired‐end reads. Variants were called using bcftools (version 1.14) in the consensus calling mode (−c). Only predicted variants with a QUAL score of ≥ 15 were retained. Bedtools subtract (version 2.27.1) was used to filter out any variants identified in potential suppressor strains that were already present in the lab *S. albus* J1074 parent strain compared to the reference sequence (NC_020990.1) and thus were accumulated independently from this experiment. The potential impact of all variants on protein‐coding sequences was assessed using snpEff (version 5.0e) (Cingolani et al. 2012). The final table was derived by manually inspecting shortlisted mutations.

### 2.4 Microscopy

For phenotypic analysis, spores were loaded onto 1.2% agarose pads prepared with TSB or 5% TSB medium, supplemented where required with 100 ng/mL anhydrotetracycline (ATc). Loading and imaging procedures were performed as previously described (de Jong et al. 2011). Samples were incubated at 30°C for 20 h prior to image acquisition to allow for germination and early mycelial development. Images were captured using a CoolSnap HQ2 camera mounted on a DeltaVision Elite inverted microscope equipped with a 100× oil immersion objective. Custom scripts developed for microcolony and fluorescence quantification are available in the Supporting Information.

For β‐glucan staining, 2 × 10^6^ CFU (estimated by OD_600_ measurement) of *S. albus* J1074 strains from freshly grown SFM agar plates were inoculated in 20 mL TSB and cultured for 20 h. Cells were centrifuged and washed with modified PEM buffer (0.4 M PIPES, 20 mM EDTA, 20 mM MgCl_2_; pH = 6.9 (KOH)), stained with lectin dye (Sigma‐Aldrich L3892) for 5 min at room temperature, washed, and resuspended in modified PEM buffer (Hasek 2006).

Cells were centrifuged as needed, loaded onto a 1.2% agarose slide prior to imaging, and examined using a Leica DM6 B fluorescence microscope equipped with a 100× objective and a DFC7000 GT camera. Images were analysed using the ImageJ software.

To determine the β‐glucan content, the mean fluorescence signal was measured at each tip (within a 20‐pixel diameter circle). A minimum of 574 data points were collected for each strain tested. Data were analysed and visualised using R software, including the ggplot2 package (Ehrlinger 2016). Statistical analysis was performed using analysis of variance (ANOVA), followed by Tukey's HSD post hoc test.

For spore morphology measurements, spores were prepared according to Kieser et al. after 7 days of growth on SFM medium. A dilution of the spore suspension was imaged on a DeltaVision Elite inverted microscope equipped with a 100× oil immersion objective and a pco.edge 4.2 sCMOS camera. Morphological dimensions were quantified using the Feret diameter, which is defined as the maximum distance between any two points along the perimeter of the spore (also known as the maximum caliper length).

### 2.5 Protein Extraction for Mass Spectrometry

For mass spectrometry, 10^7^ CFU of WT or ∆*pknB* were inoculated in 50 mL of TSB or 5% TSB and cultured for 20 h. Four biological replicates were performed under each condition. Mycelia were harvested by centrifugation (5 min, 4°C, 3000 × *g*), washed twice with phosphate‐buffered saline (PBS), and resuspended in 8 M urea buffer supplemented with 50 mM Tris, 1 mM TCEP (Roth), a protease inhibitor (Thermo Scientific), and a phosphatase inhibitor consisting of sodium molybdate (115 mM), sodium orthovanadate (100 mM), sodium tartrate dihydrate (400 mM), glycerophosphate (500 mM), and sodium fluoride (100 mM). Five millilitres of buffer was used per gram of biomass. Cell disruption was achieved via sonication (2 × 20 s, 4°C, 20 kHz), followed by centrifugation (15 min, 4°C, 3000 × *g*), and the resulting supernatant was stored at –80°C prior to subsequent steps. A detailed protocol for further sample preparation and instrumental analysis is provided in the Supporting Information.

### 2.6 Lysozyme Sensitivity and Survival Assays

Sensitivity to lysozyme was assessed by inoculating 5 μL of PBS spore suspensions and 1:10 dilutions onto TSA plates supplemented with lysozyme at a final concentration of 25 μg/mL, compared to a no‐addition control. Cultures were incubated for 3 days. Survival assays were performed similarly using TSA and 5% TSA, both supplemented or not with anhydrotetracycline at 500 ng/mL. To determine whether osmotic stress conditions induced by 5% TSA affected the lethality of the inducible DivIVA production strain (strain 65α), 0.5 M sucrose was added to the 5% TSA.

### 2.7 DivIVA‐mScarlet Production Analysis

For western blot analysis, 2 × 10^7^ CFU of *Streptomyces* strains were inoculated in 50 mL of TSB or 5% TSB, in the presence or absence of ATc (250 ng/mL), and cultured for 24 h. Up to 50 mL of cultured mycelia were harvested by centrifugation (5 min, 4°C, 3000 × *g*), washed twice with PBS, and resuspended in 1 mL PBS supplemented with a protease inhibitor (Thermo Fisher Scientific) and 1 mM DTT. Cell disruption was achieved via sonication (2 × 20 s, 4°C, 20 kHz), followed by mixing with 6× loading buffer and denaturation (15 min, 95°C). Total protein concentration was normalised and assessed using SDS‐PAGE containing 2.5% 2,2,2‐trichloroethanol (Thermo Scientific). Proteins were visualised using a stain‐free filter on a ChemiDoc (Bio‐Rad). Signal intensity was measured using ImageJ software, and the samples were adjusted accordingly. Proteins were transferred to a nitrocellulose membrane, which was blocked with a blocking solution (5% powdered milk in PBST [PBS with 0.1% Tween‐20]), then incubated with either rabbit anti‐mCherry polyclonal antibody (diluted 1:1000 in fresh blocking solution) or anti‐DivIVA_SC_ antiserum (diluted 1:5000, gift from Prof, Klas Flärdh, Lund University) (Wang et al. 2009). The membrane was washed thrice with PBST and incubated with goat anti‐rabbit IgG cross‐linked to horseradish peroxidase (Santa Cruz Biotechnology; diluted 1:5000 in fresh blocking solution), followed by three washes with PBST. The blot was incubated with SuperSignal West Pico PLUS Chemiluminescence Substrate (ThermoScientific) and visualised using a ChemiDoc (BioRad).

### 2.8 Growth Assay With Induced *cslA* Mutant

Overnight cultures were harvested by centrifugation and washed thrice with phosphate‐buffered saline (PBS). The optical density (OD) of the washed cells was adjusted to 0.1, and aliquots were plated onto 5% tryptic soy agar (TSA) plates supplemented with either 500 ng/mL of the DivIVA inducer and/or 0.5 μg/mL of thiostrepton.

## 3 Results

### 3.1 Identification of Conditions for Lethality of a DivIVA Overexpression Construct

**FIGURE 1:**
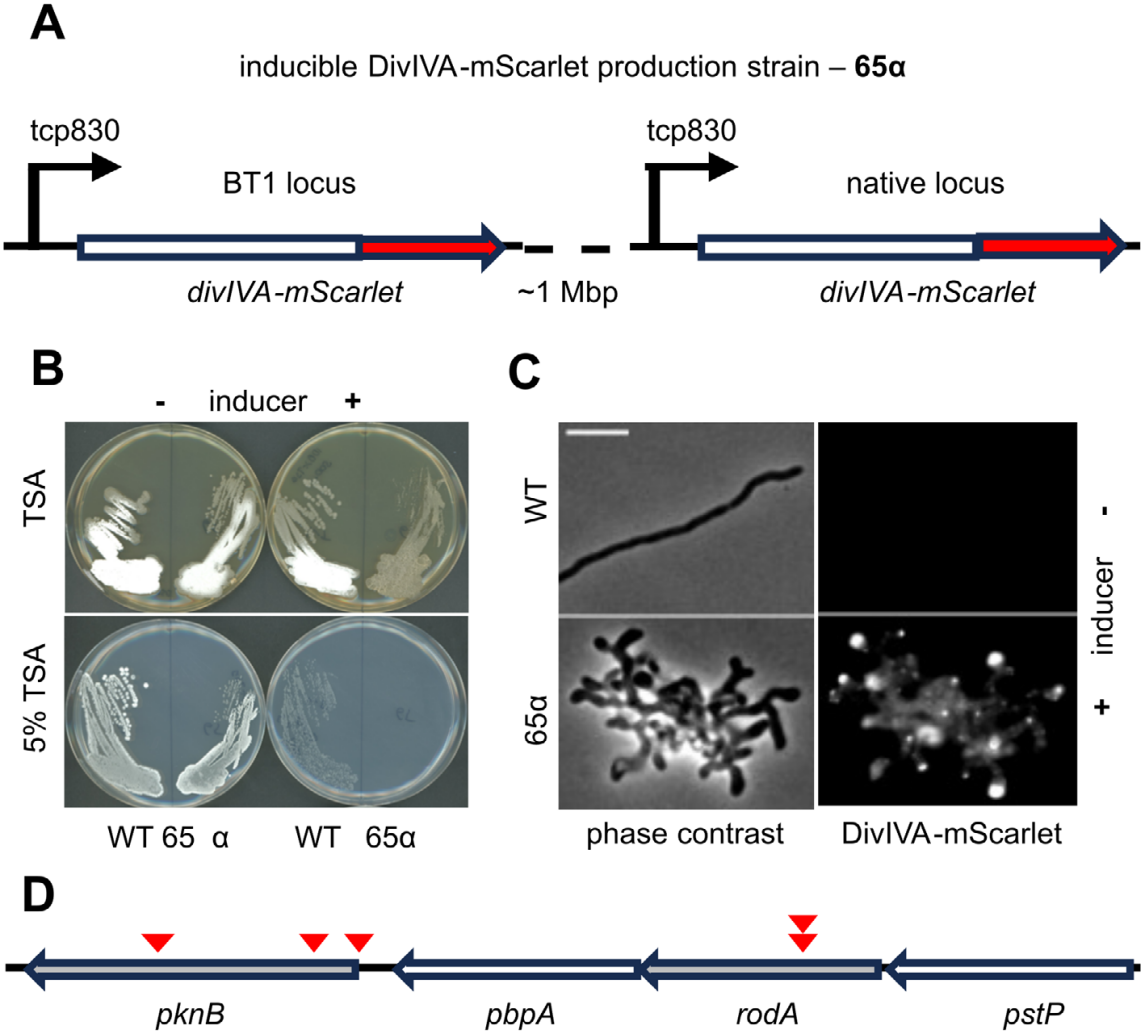
Genetic configuration and phenotype of DivIVA overproducing strain 65α (A) Pictorial representation of the genetic makeup of the *Streptomyces albus* J1074 mutant, called 65α, containing two copies of a *divIVA‐mScarlet* fusion under the control of the ATc inducible promoter tcp830. (B) Comparison of the growth behaviour of the wild‐type *Streptomyces albus* J1074 and the inducible mutant strain 65α on TSA and 5% TSA in the presence or absence of the inducer (500 ng/mL). (C) Microscopic phenotypes of *Streptomyces albus* J1074 wild‐type strain and 65α mutant grown in TSB in the presence of the inducer (250 ng/mL). Fluorescent phase‐contrast microscopy demonstrated DivIVA‐mScarlet localisation. Scale bar—5 μm. (D) Genetic organisation of the gene cluster containing *pknB* and *rodA* in *Streptomyces albus* J1074 (3,450,591‐3,457,165 on NC_020990; ~1 Mbp from BT1 locus and ~2 Mbp from *divIVA* locus). The red arrows indicate the locations of the mutations described in Table 1.

The scaffolding protein DivIVA is localised at the tip of the *Streptomyces* hyphae and is essential for regulating normal apical growth (Flärdh 2003b; Hempel et al. 2008). Previous studies have demonstrated that overproduction of DivIVA leads to hyphal tip swelling and overbranching (Hempel et al. 2008). We hypothesised that lethal overproduction of DivIVA could be suppressed by mutations in genes encoding proteins involved in tip growth and cell wall remodelling. Initially, we constructed a strain carrying a *divIVA‐mScarlet* fusion under the control of an anhydrotetracycline (ATc)‐inducible promoter located in the phiBT1 locus. Although induction of this fusion protein was non‐lethal, during the development of additional derivatives, we serendipitously created a strain, designated 65α, harbouring two inducible copies of *divIVA‐mScarlet*, one in the native locus and the other at the phiBT1 locus (Figure 1A). This strain was able to grow in the absence of the inducer, presumably because of leaky expression from the promoter (Figure 1B). However, upon induction, a strong fluorescence signal was observed at the hyphal tips, accompanied by swelling and hyperbranching (Figure 1C), aligning with previously documented observations from a DivIVA overproduction strain of *S. coelicolor* (Flärdh 2003b; Hempel et al. 2008). After testing a range of growth conditions, we found that under hypoosmotic stress conditions (1/20 reduced concentration of TSA medium “5% TSA”), the growth of strain 65α was abolished in the presence of the inducer, while it grew similarly to the wild type in its absence (Figure 1B), suggesting cell wall damage leading to osmotic lysis (rescued by 0.5 M sucrose; Figure S1).

### 3.2 A Suppressor Screen for Mutations Bypassing *divIVA* Overexpression Lethality

To identify genes potentially influencing hyphal growth, we plated *divIVA* overexpression strain 65α under the lethal conditions described above and picked the rare colonies that emerged. Of the 43 colonies from three different starting cultures, 13 mutant strains displayed robust growth under 65α lethal conditions.

Because we had two separate copies of the *divIVA* construct, we expected the suppressor mutations to lie in genes other than *divIVA*. Whole‐genome sequencing followed by bioinformatic analysis of the 13 mutants revealed apparent mutations in 122 coding regions (File S1). Table 1 lists the genes in which more than one mutation was identified. Of these, XNR_RS15060 and XNR_RS15070 were particularly interesting. They are located almost adjacent to each other in a conserved gene cluster, and their identities are apparent from the conservation of the gene cluster relative to other actinobacteria (Molle and Kremer 2010; Iswahyudi et al. 2019). We annotated these as *pknB* (XNR_RS15060) and *rodA* (XNR_RS15070) based on their homology to the equivalent operon in *S. coelicolor* (Figure 1D). In *pknB*, we identified two distinct frameshift mutations: one affecting the start codon and the other located within the kinase domain near the N‐terminus. An additional variant of this gene consisted of a single nucleotide substitution within the PASTA domain (Figure S2). While two mutations were identified in *rodA* (strains M33 and M34), both consisted of the identical L162 deletion, suggesting a single clonal origin rather than independent mutational events. In contrast, three distinct suppressor alleles were identified in *pknB* (strains M25, M40, and M43), providing stronger evidence that *pknB* loss‐of‐function is a robust and recurrent suppressor of DivIVA‐induced lethality, and prompting us to focus subsequent analysis on this gene.

**TABLE 1:** Genes recurrently mutated across independent suppressor mutants.

Gene name	Protein names	Mutant number^a^	Amino acid change
XNR_RS08380	Prolyl serine peptidase	2	M34:P63fs; M43:P63fs
XNR_RS15060	PknB	3	M25: M1fs; M40: A396D; M43: S98fs
XNR_RS15070	RodA	2	M33: L162del; M34: L162del

Abbreviations: del, deletion; fs, frame shift.

^a^ Mutant number refers to the frequency at which a mutation in a gene was recovered in a different mutant strain. The amino acid change indicates the result of a mutation in the corresponding mutant strain starting with M.

PknB kinase is an essential protein in *Mycobacterium tuberculosis* (Chawla et al. 2014) and *Corynebacterium glutamicum* (Fiuza et al. 2008), and conditional *pknB* mutations affect the maintenance of cell shape (Arora et al. 2018). PknB also phosphorylates DivIVA in Mycobacteria, but not in *Streptomyces* (Kang et al. 2005; Hempel et al. 2012). The exact role of PknB in *Streptomyces* has not been elucidated, as deletion of *pknB*, in contrast to other bacteria, has no deleterious effect, at least in wild‐type cells (Jones et al. 2011). The network of interactions described above, involving various proteins involved in cell morphogenesis, prompted us to focus on the role of PknB.

### 3.3 Deletion of *pknB* Rescues *Streptomyces* From Lethal Overexpression of *divIVA*

To test whether the mutations identified above were responsible for the suppression of the *divIVA* overexpression phenotype, we attempted to delete *pknB* in wild‐type and *divIVA* overexpression (65α) strains. As expected, deletion of *pknB* did not result in any evident phenotypes in the wild‐type *S. albus* strain, consistent with previously reported results (Jones et al. 2011). To further investigate potential developmental roles, we assessed spore morphology across three independent replicates. Quantitative analysis of spore dimensions, measured as the Feret diameter, revealed no discernible differences between the wild type and the Δ*pknB* mutant (Figure S3), and the spores produced by the mutant were morphologically indistinguishable from those of the wild type.

**FIGURE 2:**
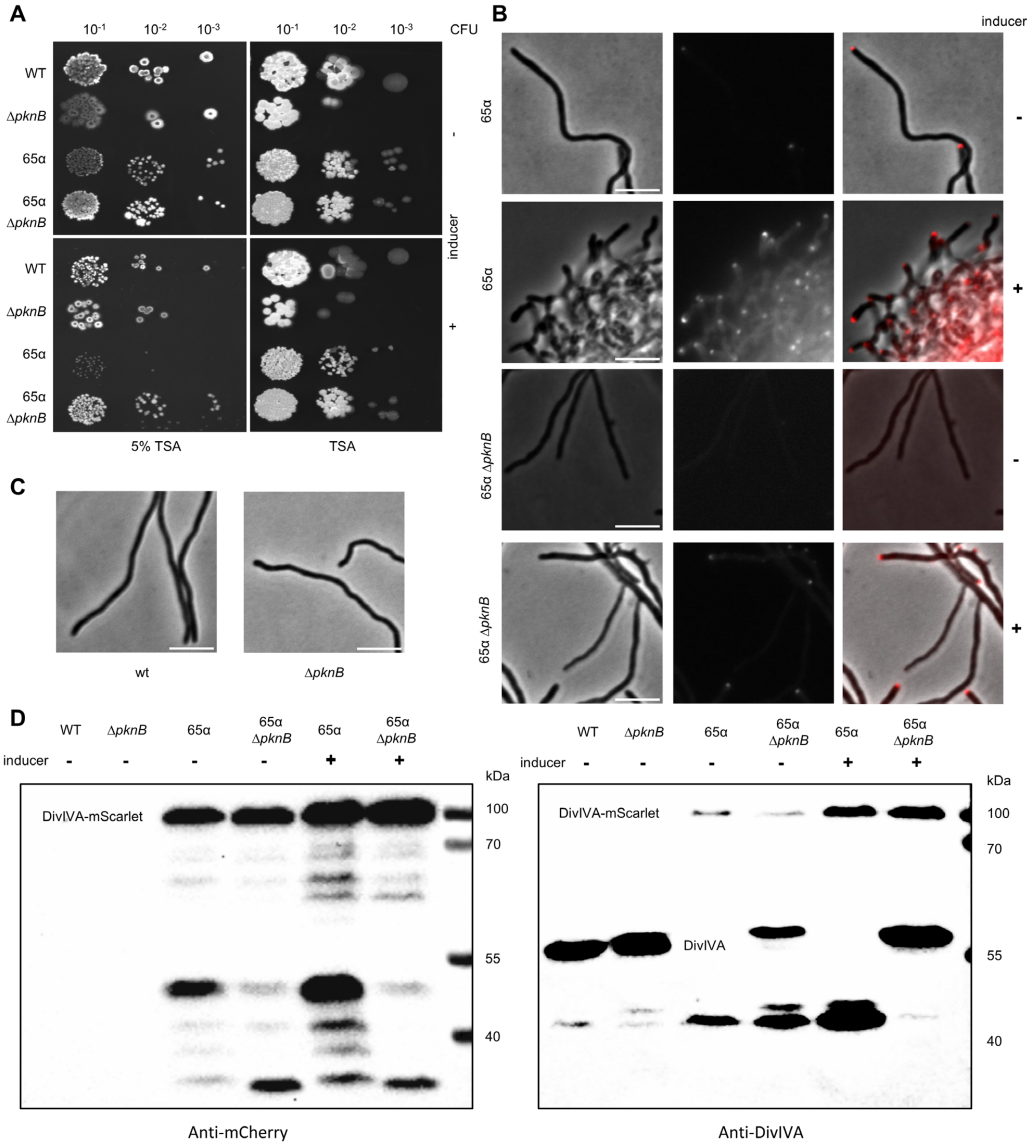
Verification of *pknB* deletion as a suppressor of lethal DivIVA overproduction. (A) Survival assay of *Streptomyces albus* J1074 strains grown on 5% TSA or TSA in the presence or absence of the inducer—ATc 500 ng/mL. (B) Phenotypes of *S. albus* J1074 strains grown in TSB in the presence or absence of the inducer ATc (100 ng mL^−1^). Fluorescence and phase‐contrast microscopy revealed DivIVA–mScarlet production. Fluorescence backgrounds and intensity thresholds were adjusted identically across samples to allow for direct comparison. In the overlay panel, fluorescence levels were adjusted for improved visualisation. Scale bar, 5 μm. (C) Phase‐contrast microscopy of *S. albus* J1074 and *S. albus* J1074 Δ*pknB* grown in TSB. (D) Western blot analysis of DivIVA‐mScarlet (theoretical mass ~68 kDa) production by *S. albus* J1074 strains grown in TSB, in the presence or absence of the inducer—ATc 250 ng/mL. Left Detection was performed using anti‐mCherry polyclonal antiserum. Right Detection was performed using anti‐DivIVA_SC_ antiserum. The molecular weight (MW) values to the right indicate the positions of the marker proteins.

However, in the 65α background deletion of *pknB* enabled growth under otherwise lethal conditions (5% TSA + ATc) (Figure 2A). Microscopic imaging (Figure 2B,C, quantification Figure S4) revealed that the 65α strain had an increased frequency of branching compared with the wild type, even in the absence of the inducer, while in the presence of ATc, it exhibited hyperbranching, bulging, and an accumulation of mScarlet at the hyphal tips. In contrast, the 65α ∆*pknB* strain exhibited reduced branching and a diminished fluorescent signal compared with the 65α parent. Indeed, 65α ∆*pknB* mycelia closely resembled those of the wild‐type strain, even in the presence of ATc. Although we could still detect a fluorescent signal from mScarlet, the fluorescence was notably reduced compared to that of 65α. In the absence of the inducer, most hyphae (67%) exhibited a diffuse cytoplasmic distribution of the mScarlet signal. Upon induction, this proportion decreased to approximately 33%, with the remaining hyphae displaying a clear accumulation of fluorescence at the tips of the hyphae.

Sanger sequencing of both *divIVA‐mScarlet* fusion genes did not reveal any mutations. Presumably, the lowered signal at the hyphal tips could be a result of DivIVA‐mScarlet being distributed more widely throughout the hyphae or diminished protein levels due to decreased synthesis or increased degradation.

To compare the total DivIVA protein levels between strains, we analysed DivIVA–mScarlet abundance in 65α and 65α *ΔpknB* by Western blotting using anti‐mScarlet‐ and anti‐DivIVA‐ antibodies (Figure 2D; loading control in Figure S5). Native DivIVA (predicted 41.1 kDa) migrated at approximately 60 kDa in both wild‐type and Δ*pknB* backgrounds. Its abundance was not significantly altered in the absence of *pknB*. The upward shift in the apparent molecular weight likely reflects dimerisation or altered electrophoretic mobility. In the 65α background, the DivIVA–mScarlet fusion protein (predicted 68 kDa) migrated near 100 kDa. Upon ATc induction, the intensity of the full‐length fusion increased strongly when detected by both the anti‐DivIVA and anti‐mScarlet antibodies, confirming that induction elevated total DivIVA–mScarlet levels. Both *65α* and *65α ΔpknB* strains displayed additional faster‐migrating species. These fragments were detected by anti‐DivIVA but not by anti‐mScarlet antibody, indicating that they represent N‐terminal DivIVA cleavage products lacking the fluorescent tag. Conversely, the anti‐mScarlet blot revealed smaller mScarlet‐containing fragments not recognised by anti‐DivIVA, consistent with C‐terminal cleavage events. ATc induction increased the abundance of these fragments in *65α*, consistent with the higher overall expression of the fusion protein.

Loss of *pknB* did not reduce the amount of full‐length DivIVA–mScarlet; however, it markedly altered the pattern of degradation products. The 65α *ΔpknB* strain accumulated several distinct DivIVA‐sized fragments, suggesting that *pknB* influences the stability or processing of the DivIVA–mScarlet fusion protein indirectly. This raises the possibility that PknB‐dependent signalling influences the proteolytic turnover of DivIVA, potentially serving as a mechanism to control DivIVA levels under conditions of overexpression.

### 3.4 Deletion of *pknB* Causes Hyperphosphorylation of CslA

The kinase PknB has been reported to be involved in various processes across various organisms, including cell division, peptidoglycan synthesis, protein synthesis, and stress responses (Jones et al. 2011; Dworkin 2015; Richard‐Greenblatt and Av‐Gay 2017). To better understand its role in *S. albus*, we analysed the phosphoproteomes of wild‐type and *∆pknB* *S. albus* strains, with particular focus on proteins implicated in polar growth. The results identified 27 phosphorylated proteins, of which three were significantly hypophosphorylated in the *pknB* mutant, while one was significantly hyperphosphorylated (Table 2 and File S2; proteomics data are available in File S3). Among the expected candidates, we anticipated changes in DivIVA phosphorylation, as well as in other regulators such as the kinase AfsK or the phosphatase SppA, which is known to dephosphorylate DivIVA (Hempel et al. 2012; Passot et al. 2022). Although SppA phosphorylation was indeed altered in the Δ*pknB* background, no corresponding changes in DivIVA phosphorylation were detected in our experimental setup, and this pathway was therefore not investigated further. Our attention was drawn to CslA, a synthase previously associated with polar growth, which was hyperphosphorylated on residue T17 in the Δ*pknB* background, making it a compelling candidate for mediating PknB's effects on hyphal development. This protein localises at hyphal tips and is responsible for the production of a beta‐glucan‐containing polysaccharide that is thought to provide additional physical protection, potentially required for the continuous remodelling of the growing cell wall (Xu et al. 2008). Loss of this polymer impairs the morphological development and stability of the cell wall (Xu et al. 2008; Zhong et al. 2022, 2024). CslA forms a protein complex with the copper radical oxidase GlxA, and it is a known interaction partner of DivIVA in *S. coelicolor* (Xu et al. 2008; Zhong et al. 2022, 2024).

**TABLE 2:** Significantly different protein phosphorylation patterns of *Streptomyces albus* J1074 WT and ∆*pknB* strains grown in TSB medium.

locus_tag	Protein name	Phosphorylation in ∆*pknB* vs WT
XNR_RS17030	FolK	Hypophosphorylated ↓
XNR_RS11880	GroL	Hypophosphorylated ↓
XNR_RS08650	CslA	Hyperphosphorylated ↑
XNR_RS14580	SppA	Hypophosphorylated ↓

**FIGURE 3:**
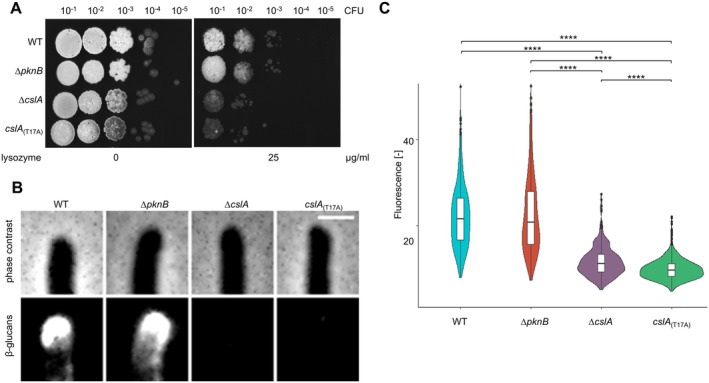
Impact of mutations in *cslA* on glucan production (A) Lysozyme sensitivity of *Streptomyces albus* J1074 strains grown on TSA. (B) Phenotypes of *S. albus* J1074 strains grown on TSA. Cells were stained with lectin dye to visualise β‐glucans. Scale bar—1 μm. (C) Quantification of mean β‐glucan production at the tip by *S. albus* J1074 strains grown in TSB. The width of each plot indicates the number of collected data points for a specific fluorescence. Significant differences, according to ANOVA followed by Tukey's HSD post hoc test, are marked with ****p ≤ 0.0001.

To evaluate the effect of phosphorylation on the function of CslA, we constructed a strain producing phosphoablative CslA (*cslA*_T17A_) and a deletion mutant. We confirmed the production of the phosphoablative ClsA using a proteomics experiment (Figure S6). Previous studies have shown that deletion of *cslA* increases sensitivity to lysozyme (Zhong et al. 2022); we therefore tested whether preventing phosphorylation at T17 would similarly affect cell wall integrity. Both *cslA* mutants displayed hypersensitivity to lysozyme, suggesting that phosphorylation is important for CslA activity (Figure 3A). To determine whether the reciprocal change—hyperphosphorylation of CslA in the Δ*pknB* background—had the opposite effect, we assessed lysozyme sensitivity of the *pknB* deletion strain. This strain responded to lysozyme similarly to the wild type, indicating that hyperphosphorylation of CslA alone is not sufficient to further enhance cell wall protection beyond wild‐type levels. Next, we used a lectin dye to stain β‐glucans (Figure 3B,C) and found that the signal at the tips of hyphae was completely abolished in the *cslA* knockout strain, similar to previously reported data on a *cslA* deletion strain (Xu et al. 2008; Zhong et al. 2022). Again, the strain producing the phosphoablative variant of CslA (*cslA*_(T17A)_) behaved similarly to the deletion strain when stained for β‐glucans, indicating that phosphorylation is likely required for the β‐glucan synthetic activity of CslA. Overall, these data indicate that phosphorylation plays an important role in β‐glucan production, although the role of PknB in this process is likely to be indirect.

### 3.5 Overexpression of CslA Rescues Cells From DivIVA Overproduction Lethality

**FIGURE 4:**
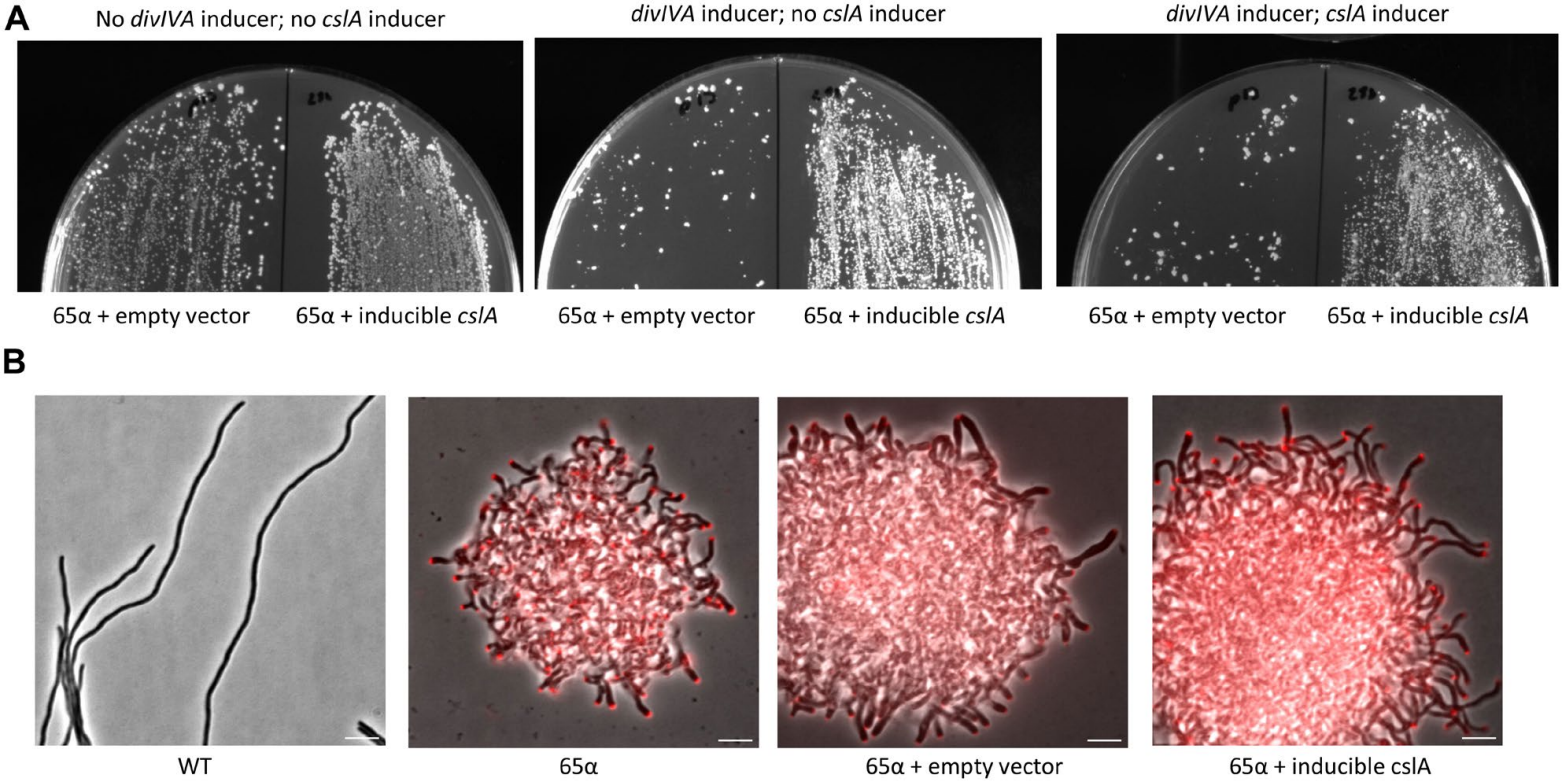
Overexpression of *cslA* rescues growth under conditions of DivIVA overproduction (A) Growth of 65α attBϕC31::pIJ6902 (empty plasmid) and 65α attBϕC31::pBK288 (*cslA* under *tipA* inducible promoter) grown on 5% TSB plates in the presence or absence of an inducer for DivIVA or CslA. (B) Phenotypes of *Streptomyces albus* J1074 strains (WT, 65α, 65α attBϕC31::pIJ6902 [empty plasmid], and attBϕC31::pBK288 expressing *cslA* under the *tipA*‐inducible promoter) grown in 5% TSA medium in the presence of anhydrotetracycline (100 ng mL^−1^). DivIVA–mScarlet production was visualised by fluorescence microscopy and is shown as a red signal overlaid on phase‐contrast images. Fluorescence background and intensity thresholds were adjusted identically across all samples to allow for direct comparison.

The results described above, showing that phosphorylation of CslA is likely required for the β‐glucan synthetic activity, suggest that the deletion of *pknB* might increase the activity of CslA, via increased phosphorylation albeit indirectly. If so, *cslA* overexpression might also rescue cells from *divIVA‐mScarlet* overexpression toxicity. To test this, a plasmid carrying a thiostrepton‐inducible copy of *cslA* (under *tipA* promoter) was introduced into the 65α background, with an empty plasmid as a control. In the absence of DivIVA overproduction, both strains grew well on 5% TSA. However, when *divIVA* was induced with ATc, the strain carrying the *cslA* plasmid showed significantly improved growth compared to the empty‐plasmid control. This occurred even without thiostrepton induction of the second *cslA* copy. When both *divIVA* and *cslA* were induced together (ATc + thiostrepton), growth was similar to conditions without *cslA* induction, suggesting that even leaky expression from the *tipA* promoter was sufficient to rescue the *divIVA*‐induced growth defect (Figure 4A) (Tong et al. 2019). Unexpectedly, *cslA* induction did not restore the overbranching phenotype associated with *divIVA* overexpression, indicating that increased CslA only partially suppressed the DivIVA overexpression phenotype (Figure 4B, quantification Figure S4). Plausibly, its contribution to viability may involve the enhancement of the protective layer at the cell tip.

## 4 Discussion

**FIGURE 5:**
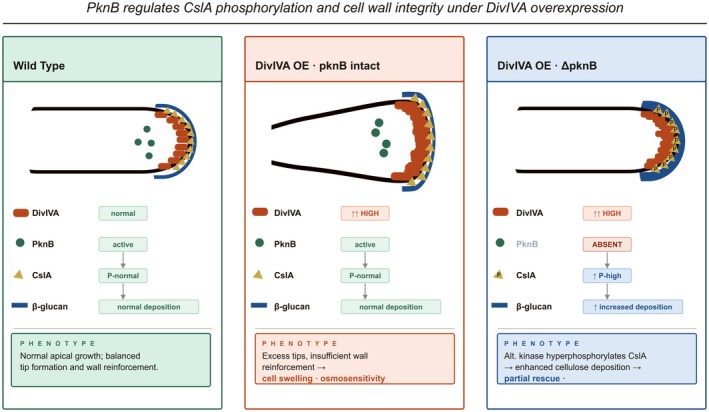
Proposed model of how phosphorylation regulates cell wall stability in *Streptomyce*s, including the proposed roles of the kinase PknB and the cellulose synthase CslA.

To identify the factors required for tip growth and cell wall integrity, we performed a suppressor screen based on the lethal overexpression of *divIVA*, a key polarity determinant. Under these conditions, spontaneous suppressor mutants arise at low frequencies, and whole‐genome sequencing revealed recurrent mutations in the Ser/Thr kinase gene *pknB*. PknB is a conserved Ser/Thr kinase found across actinobacteria, where it has been implicated in the regulation of cell division, cell wall synthesis, and stress responses. Targeted deletion of *pknB* in the DivIVA overexpression background confirmed that the loss of this kinase is sufficient to suppress lethality (Figure 5), indicating that PknB activity becomes problematic when DivIVA‐driven tip formation is excessive.

Because PknB has been implicated in diverse cellular processes, we used global phosphoproteomics to define its downstream targets. This analysis revealed that the cellulose synthase CslA was hyperphosphorylated in the absence of PknB. To determine the functional relevance of this modification, we generated both a *cslA* deletion mutant and a phosphoablative T17A variant. Substitution of T17 with alanine abolished CslA activity, as shown by quantitative microscopy and increased lysozyme sensitivity, demonstrating that phosphorylation at this residue is essential for its proper function. The identification of CslA as a PknB‐dependent target provides a new perspective on the regulation of β‐glucan biosynthetic complexes in Actinobacteria. CslA and GlxA form a functional unit, and the coordination between the glycosyltransferase (CslA) and its auxiliary oxidase (GlxA) is likely critical for proper polymer secretion and hyphal reinforcement (Xu et al. 2008; Zhong et al. 2022, 2024). Given that T17 is located in the cytoplasmic N‐terminus of CslA, we propose that PknB‐dependent signaling acts as a regulatory switch that influences the activity or organization of the CslA/GlxA complex. The CslA phosphoablative T17A variant fails to support β‐(1,4)‐glucan synthesis. This suggests that without phosphorylation, the complex may fail to reach an active conformation. Such coordination allows the cell to rapidly match cell wall reinforcement with the mechanical requirements of the hyphal tip, particularly under rapid extension or DivIVA‐induced stress. The single phosphorylation site at CslA contrasts sharply with the phosphorylation pattern of the polarity determinant DivIVA, which in *S. coelicolor* is modified by the kinase AfsK at five distinct residues (T304, S309, S338, S344, and S355) (Saalbach et al. 2013). While the multi‐site phosphorylation of DivIVA is believed to allow for graded or redundant regulatory responses that modulate hyphal branching, the single‐site modification of CslA at T17 implies a more direct, potentially binary control mechanism. Consistent with this, *cslA* overexpression partially rescued the growth defect of the DivIVA overproduction strain, although it did not fully restore normal growth and overbranching was still detected. This indicates that while CslA is important for maintaining cell wall integrity of overbranching hyphae, additional PknB‐dependent targets must also contribute to the altered physiology observed in Δ*pknB* mutants.

These findings identify PknB as a central regulator of cell wall homeostasis that becomes detrimental when tip formation is elevated by DivIVA overproduction (Figure 5). Furthermore, they implicate CslA phosphorylation as a key downstream mechanism required to maintain cell wall integrity under these conditions. The observation that CslA is more phosphorylated in the *pknB* mutant suggests that PknB modifies the activity of enzyme(s) that control phosphorylation of CslA. One plausible model is that PknB represses a phosphatase or activates a second kinase, such that an alternative kinase hyperphosphorylates CslA in the absence of PknB. In *Streptomyces*, several Ser/Thr kinases, including AfsK, and the phosphatase SppA have established roles in controlling cell polarity and cell wall–associated substrates, including DivIVA. AfsK is particularly noteworthy as it is a non‐essential kinase already known to regulate polar growth and hyphal branching by modifying DivIVA (Hempel et al. 2012). Given that *Streptomyces* encodes a large repertoire of at least 34 Ser/Thr kinases, it is possible that PknB acts as a high‐level regulator that normally restricts the activity of one or more of these alternative kinases at the hyphal tip.

Interestingly, SppA was hypophosphorylated in our global phosphoproteome. Since SppA is known to dephosphorylate DivIVA, the reduced phosphorylation in the Δ*pknB* background might be expected to result in altered DivIVA phosphorylation (Passot et al. 2022). However, no corresponding changes in DivIVA phosphorylation were detected in our experimental setup, suggesting that this pathway does not play a major role under the conditions tested, or that compensatory mechanisms maintain DivIVA phosphorylation levels. The absence of changes in DivIVA phosphorylation may be due to the fact that in *Streptomyces*, DivIVA phosphorylation is primarily and robustly mediated by the kinase AfsK (Hempel et al. 2012). It is plausible that AfsK activity is sufficient to maintain stable DivIVA modification levels, effectively compensating for any subtle PknB‐dependent changes in SppA activity. Nevertheless, the altered phosphorylation state of SppA in the absence of PknB suggests that SppA's regulatory activity is itself under PknB control. This raises the intriguing possibility that the substrate specificity of SppA—or its overall phosphatase activity—could extend beyond DivIVA to include CslA. If PknB is required to maintain SppA in an active, phosphorylated state, the loss of PknB would lead to a decrease in SppA activity, providing a mechanistic explanation for the observed hyperphosphorylation of CslA. Whether SppA or another as‐yet‐unidentified enzyme mediates CslA phosphorylation downstream of PknB remains to be determined. Thus, PknB likely acts upstream of a broader regulatory network that coordinates multiple enzymatic activities to ensure the stability of the cell envelope during rapid tip extension.

Finally, the DivIVA overexpression phenotype provides a physiological context for the observed regulatory circuit. Excess DivIVA generates numerous closely spaced tips, creating multiple mechanically vulnerable sites that require rapid reinforcement. The severe growth defect and pronounced low osmolarity sensitivity of the DivIVA overproduction strain are consistent with a weakened cell envelope that is unable to withstand turgor pressure when tip reinforcement is insufficient. In this scenario, CslA becomes particularly important, and its higher levels and correct phosphorylation of CslA are likely required to deposit β‐glucan at all nascent growth zones. The partial suppression of the DivIVA overexpression phenotype by *cslA* overexpression fits naturally within this model; however, the failure of CslA overexpression to restore normal growth in the Δ*pknB* background indicates that multiple PknB‐dependent pathways are required to maintain cell wall homeostasis when the tip number is increased.

In summary, using *divIVA* overexpression as a perturbation of tip‐associated cell wall synthesis allowed us to identify PknB as a central regulator of cell wall homeostasis, acting through a phosphorylation network that ultimately controls CslA‐dependent reinforcement of newly formed tips, but also involving additional PknB‐regulated factors that remain to be identified.

## Author Contributions

**Marta Derkacz:** investigation, visualization, writing – original draft. **Andrew Watson:** investigation. **Akshada Gajbhiye:** investigation. **Michał Tracz:** investigation, visualization. **Dagmara Jakimowicz:** writing – review and editing. **Matthias Trost:** supervision. **Jeff Errington:** conceptualization, funding acquisition, writing – review and editing, supervision. **Bernhard Kepplinger:** funding acquisition, supervision, writing – review and editing, conceptualization, investigation, project administration, visualization, writing – original draft.

## Funding

This work was supported by Narodowe Centrum Nauki, 2020/39/D/NZ1/00303, Wellcome Trust, 209500.

## Disclosure

Copyright statement: All figures and tables presented in this manuscript are original works created by the authors. No copyrighted, trademarked, or previously published material has been reproduced, and therefore no copyright permissions were required.

## Ethics Statement

The authors have nothing to report.

## Conflicts of Interest

The authors declare no conflicts of interest.

## Supporting information


**Table S1:** *Streptomyces albus* J1074 strains used in this study.
**Table S2:** Escherichia coli strains used in this study.
**Table S3:** Plasmids used in this study.
**Table S4:** Oligonucleotides used for cloning in this study.
**Figure S1:** Survival assay of S. albus J1074.
**Figure S2:** Alphafold model of PknB with the mutations in the suppressor screen labelled.
**Figure S3:** Spore morphology analysis.
**Figure S4:** Quantification of microcolonies and single‐cell fluorescence.
**Figure S5:** Stain free loading gel control.
**Figure S6:** Volcano plot of differential protein expression in S. albus J1074 versus cslA(T17A).

**File S1:** Full list of mutations from suppressor screen.

**File S2:** Mass spectrometry phosphoproteomics data.

**File S3:** Mass spectrometry proteomics data.

**File S4:** Mass spectrometry analysis (CslA analysis).

## Data Availability

The genome sequencing data that support the findings of this study are openly available in NCBI at https://www.ncbi.nlm.nih.gov/sra (accession number PRJNA1099611). The mass spectrometry proteomics data have been deposited to the ProteomeXchange Consortium via the PRIDE (Perez‐Riverol et al. 2022) partner repository with the dataset identifier PXD053764 and PXD071342.
